# The Human Health Risk Assessment of Heavy Metals Impurities (Cd and Pb) in Herbal Medicinal Products as *Menthae piperitae tinctura* (*Mentha × piperita* L., folium) Available in Pharmacies from Poland

**DOI:** 10.3390/toxics10050273

**Published:** 2022-05-23

**Authors:** Kamil Jurowski, Mirosław Krośniak

**Affiliations:** 1 Institute of Medical Studies, Medical College, Rzeszów University, Al. mjr. W. Kopisto 2a, 35-959 Rzeszow, Poland; 2 Department of Food Chemistry and Nutrition, Medical College, Jagiellonian University, Medyczna 9, 30-688 Krakow, Poland; miroslaw.krosniak@uj.edu.pl

**Keywords:** HM—heavy metals, HMP—herbal medicinal product, ICH Q3D—International Council for Harmonization of Technical Requirements for Pharmaceuticals for Human Use, PDE—permitted daily exposure, HHRA—human health risk assessment

## Abstract

Appropriate human health risk assessment (HHRA) is desire in modern regulatory toxicology, especially for elemental impurity studies. The aim of this article is the comprehensive HHRA of two heavy metals impurities—Cd and Pb in herbal medicinal products (HMP) as *Menthae piperitae tinctura* (*Mentha × piperita* L., folium) available in Polish pharmacies. These phytopharmaceuticals registered in EU are very common and usually applied OTC products by adults and also children/adolescents. For this purpose, we applied double regulatory approach, including: (1) requirements of ICH Q3D R1 guideline about elemental impurities and (2) additionally margin of exposure (MoE)-based concept to cover also specific population groups. Raw results shows that Cd and Pb were present in all analyzed HMP with *Mentha × piperita* L., folium (PTM1–PTM10) available in Polish pharmacies. In all samples, Cd impurities (in the range: 0.305–0.506 µg/L) were greatly lower than Pb impurities (in the range: 1.122–4.4921 µg/L). The HHRA of Cd and Pb impurities considering ICH Q3D R1 guideline-based approach made it possible to conclude that all results were below the permissible limit set by FAO/WHO for medicinal herbs and plants in different countries (300 µg/kg for Cd and 10,000 µg/kg for Pb). Additionally, the estimated daily intake of investigated elemental impurities compared to the PDE value confirm all samples safety. The second approach, an MoE-based strategy, indicated that the obtained values of MoE for Cd and Pb in daily dose for each samples were above 10,000; hence, exposure to these elemental impurities would not cause a health risk for all investigated population groups (children, adolescents, and adults). To the best our knowledge, this article is the first study about heavy metals impurities level in final HMPs as *Menthae piperitae tinctura* (*Mentha × piperita* L., folium) available in Polish pharmacies.

## 1. Introduction

There is no doubt that cadmium and lead are the most hazardous elements for human health. These heavy metals (and Hg with As) belong to the first class of elements to be considered in the risk assessment of final pharmaceutical products considering recommendations of the ICH Q3D R1 (International Council for Harmonization of Technical Requirements for Pharmaceuticals for Human Use) guideline about elemental impurities in final pharmaceuticals [1]. These elements are defined as first class (most important from toxicological point of view) because they are human toxicants that have limited or no applications in the manufacture process in pharmaceutical industry. Due to their toxic properties, these elements require comprehensive assessment. Hence, they can be considered only as impurities because their occurrence in final pharmaceutical products usually comes from:

- Commonly applied materials [1],
- Organic reagents [2],
- Trace amounts of metal catalysts [3,4],
- Environmental pollutions in raw materials (e.g., plant extracts) [5,6].

Particularly, the last source of contaminations, environmental pollutions in raw materials, are the potential and most important sources of cadmium and lead in finished pharmaceutical products containing raw plant materials. In the European Union (EU), an especially important term is “herbal medicinal product” (HMP), which should be understood as “any medicinal product, exclusively containing as active ingredients one or more herbal substances or one or more herbal preparations, or one or more such herbal substances in combination with one or more such herbal preparations” [7]. These products are very popular among the European population, in particular, as it concerns children (8–12 years old) and adolescents (12–18 years old) because (1) there is a false belief that products of natural origin are better and healthier [8], and (2) there is a widespread belief that consumers are usually convinced that products available in pharmacies are better than those available in the market [9]. Therefore, such products may pose a potential risk due to the possible heavy metals impurities to patients who apply HMP because such phytopharmaceuticals are used usually for a long time period, often, and in relatively high doses [10]. Moreover, there are common opinions among the population (especially in Poland) about the alleged heavy metals impurities in HMP available in pharmacies without any scientific background.

An example of an extremely popular HMP in Poland is a tincture with *Menthae piperitae tinctura* (*Mentha × piperita* L., folium). It should be noted that *Mentha × piperita* L. is believed to be a hybrid of spearmint (*Mentha spicata* L.) and water mint (*Mentha aquatica* L.) and belongs to the family Labiatae (*Lamiaceae*) [11]. This phytopharmaceutical is usually available in pharmacies as a preparation in liquid dosage forms (tincture) for oral use in the specified indication exclusively based upon long-standing use [12]. This phytopharmaceutical as a tincture (officially—*Menthae piperitae tincture*; *Mentha × piperita* L., folium) is officially registered as HMP in the EU [13] and is applied for the treatment of symptomatic relief of digestive disorders such as dyspepsia and flatulence [12]. The posology is different but very similar depending on the manufacturer and country [13]:

- Children 8–12 years: 8–10 drops maximum 2–3 times per day;
- Adolescents 12–18 years: 10–15 drops maximum 3 times per day;
- Adults: 15–20 oral drops 3 times per day.

Mentioned data about posology is extremely important from a human health risk assessment (HHRA) point view because they form the point of reference for the estimation of daily exposure. However, the earlier-mentioned ICH Q3D R1 guideline about elemental impurities [1] in final pharmaceuticals indicates a strategy for comparing estimated values of daily exposure of each elemental impurities with corresponding permitted daily exposure (PDE) values but for the entire population (without distinguishing between children and adults). As is known from contemporary literature, children should not be considered as small adults [14]; hence, for comprehensive HHRA, another appropriate toxicological approach should be applied. It seems that very useful in this situation can be the concept of margin of exposure (MoE) [15]. MoE can be defined as the ratio between a point of departure (POD_sys_; usually historical NOAEL or BMDL_10_ values from oral studies; mg/kg bw/day) and estimated exposure (EE, mg/kg bw/day): MoE = {POD_sys_/EE}. This concept is very universal and useful and can be used for impurities that are both genotoxic and carcinogenic, irrespective of their origin [16]. In general, an MoE of 10,000 or higher, if it is based on the BMDL_10_ from an animal carcinogenicity study and considering overall uncertainties in the interpretation, would be of low concern from a public health point of view and might be reasonably considered as a low priority for risk management actions. Hence, the value of MoE of 10,000 (or higher) is considered of low concern from a public health point of view with respect to the carcinogenic effect [16]. This concept is usually applied for food safety assessment; however, it is also applied in literature for different herbal products (e.g., [17]). Therefore, it will be suitable as an additional regulatory approach for HHRA of heavy metals in HMP because, in this approach, children and adults can be treated separately.

Based on actual critical literature review, there are a great deal of articles about the determination of heavy metals in different biological samples and food products; however, there is a lack of comprehensive toxicological studies about HHRA related to heavy metals impurities in HMP including both the requirements of the ICH Q3D R1 guideline and the MoE-based concept including children and adolescents separately from adults in the estimated exposure step. Hence, the aim of our study is not determination of chosen heavy metals (Cd and Pb) but the comprehensive HHRA of these impurities in HMP as *Menthae piperitae tinctura* (*Mentha × piperita* L., folium) available in Polish pharmacies. The justifications for choosing these two metals were:

(1) The fact that Cd and Pb impurities in final pharmaceuticals are most important from toxicological point of view (first class of elemental impurities in ICH Q3D R1 guideline [1]);
(2) Our analytical possibilities and scientific experiences [18,19,20];
(3) The possibility of this kind of contamination in the raw plant materials used for the production of these HMP based on literature review [21,22];
(4) The rarity of this type of research in the field of comprehensive HHRA of elemental impurities in final pharmaceutical products;
(5) The need to fill a gap in the scientific literature regarding the control of these elemental impurities in phytopharmaceutical product.

In our studies, we analyzed *Menthae piperitae tinctura* (*Mentha × piperita* L., folium) available in Polish pharmacies (*n* = 10); all phytopharmaceuticals were registered in the EU as HMP. The choice of this product was dictated by the fact that these HMP are very popular in Poland based on: (1) the results of physicians’ and pharmacists’ opinions (*n* = 40) from Kraków and Rzeszów; (2) interview about popularity among patients (*n* = 35; 12–42 years old) from Niepołomice and Rzeszów (Poland); and (3) literature review, including pharmaceutical websites and blogs in Poland (accessed on April 2022).

It should be noted that there exist a few modern analytical techniques commonly applied in determination of elements, especially inductively coupled plasma-mass spectrometry (ICP-MS), neutron activation analysis (NAA), or X-ray fluorescence-based technique (XRF); however, in our studies, we used the well-known and accepted AAS technique with flame atomization (F-AAS) due to our instrumental possibilities but also its simplicity, availability, and well-grounded analytical background. Moreover, we confirm that HHRA studies do not require application of the advanced analytical techniques.

It should be emphasized (as mentioned earlier) that the purpose of the research was not the determination of Cd and Pb in HMP as tinctures with *Mentha × piperita* L., folium; hence, the focus was not on innovations in determination but on the strategies/approaches in comprehensive HHRA related to Cd and Pb impurities, including both the requirements of the ICH Q3D R1 guideline and the MoE-based concept including children and adolescents separately from adults in estimated exposure step.

## 2. Materials and Methods

### 2.1. Samples

The subject of the study was *Menthae piperitae tinctura* (MPT) purchased from pharmacies in Poland (Niepołomice, Kraków and Rzeszów) in the period from January 2022–March 2022. In our studies, we considered only pharmaceutical products registered in the EU. It should be emphasized that we analyzed all available HMP with *Mentha × piperita* L., folium in Poland (*n* = 10). It should be noted that only a few independent manufacturers produce these kinds of pharmaceutical products in Poland, hence the relatively low number of available samples. All of the considered MPT belong to over-the-counter medicines. To maintain the highest methodological standards, all samples were coded as (MPT1–MPT10). The summary of all analyzed samples is shown in Table 1.

### 2.2. Samples Preparation, Determination of Cd and Pb, Analytical Calibration, Quality Control, and Data Processing

The samples were digested using a microwave oven MDS 2000 (CEM USA). Firstly, all HMP with *Mentha × piperita* L. folium were homogenized. Then, approximately 0.5 mL of each sample (0.5 mL samples containing ethyl alcohol were taken to dryness before digestion) were placed into microwave digestion tube (Teflon vessels) and digested (1 h) with 5.0 mL of concentrated nitric acid (HNO_3_, 63%). After this step, all samples were cooled to a room temperature (20 °C). The final volume was 20 mL. For all samples to increase the precision of the results, five replications were performed.

During the determination operation, the Perkin–Elmer 5100 ZL atomic absorption spectrometer (AAS) with a graphite furnace was applied to measure the total contents of Cd and Pb each sample. It should be noted that for all steps during analytical operations, we used demineralized water (ultrapure demineralized water had been obtained by Milli-Q) and nitric acid (65%) of spectroscopic grade (Merck SupraPur, Darmstadt, Germany). The applied instrumental conditions for Cd and Pb determination by AAS technique was summarized in Table 2.

For analytical purposes, appropriate standard solutions were applied. For cadmium, standard solution traceable to SRM from NIST–Cd(NO_3_)_2_ in 0.5 mol/L HNO_3_, 1 g/L Cd CertiPUR^®^, catalog product: 1.19777.0500); for Pb, standard solution traceable to SRM from NIST–Pb(NO_3_)_2_ in 0.5 mol/L HNO_3_ and 1000 mg/L Pb CertiPUR^®^ (catalog product: 1.19777.0500) were prepared by dilution of certified standard solutions and 1 mg/L MERC of corresponding metal ions. The purchased certified reference material (CRM; BCR-482; IRMM, Belgium) was material prepared from lichen. Additionally, the second certified reference material (Corn Flour, INCT-CF-3) was purchased from the Institute of Nuclear Chemistry and Technology—Department of Analytical Chemistry (Warsaw, Poland).

For the analytical calibration step, we obtained a calibration function that includes the following levels: 0.0, 0.5, 1.0, and 2.0 µg/L for Cd and 0.0, 1.0, 2.0, 5.0, and 10.0 µg/L for Pb. The obtained values of correlation coefficients (R) were 0.998 and 0.997 for Cd and Pb, respectively.

For quality control, we obtained the following values for recoveries: 98.7% and 98.5% for Cd and Pb, respectively. The calculated limits of quantification (LOQs) were 0.34 µg/L for Cd and 0.97 µg/L for Pb. The calculated limits of detection (LODs) were 0.16 µg/L for Cd and 0.46 µg/L for Pb.

Data were analyzed, and graphs were plotted using statistical software Origin 2021 Pro the Ultimate Software for Graphing and Analysis (OriginLab Corporation, One Roundhouse Plaza, Suite 303, Northampton, MA 01060, USA) licensed by the Jagiellonian University in Krakow. Data processing and all basic descriptive calculations, compilation, and storage of obtained data at laboratory stage were made using Excel 2010 (Microsoft Office). The resultant data of five independent replicates are expressed as the mean ± standard error.

### 2.3. The Applied Strategies in Comprehensive Human Health Risk Assessment (HHRA) of Cd and Pb Impurities in HMP with Mentha × piperita L., folium

As was mentioned earlier, for assessment of elemental impurities in final pharmaceuticals, the strategy based on ICH Q3D R1 guideline about elemental impurities [1] is very useful. This strategy was successfully applied in our previously published studies [23,24,25,26]. The idea of this strategy is an estimation of single-dose exposure and daily-dose exposure for each element in all samples and then comparison of obtained results (estimated values of daily exposure of each elemental impurities) with corresponding permitted daily exposure (PDE) recommended by ICH Q3D R1 guideline [1]. However, considering the fact that the investigated phytopharmaceuticals are also applied in treatment of children and adolescents (based on posology from each manufacturer—see Table 1), an additional strategy is needed. 

For conducting comprehensive HHRA (covering children and adolescents), we applied an additional MoE-based strategy [15]. MoE can be defined as the ratio between a point of departure (PODsys; usually historical NOAEL or BMDL_10_ values from oral studies) and an estimate of the estimated exposure—Equation (1):

MoE = POD_sys_/EE (1)

where:

POD_sys_—point of departure (e.g., mg/kg bw/day);

EE—estimated exposure (e.g., mg/kg bw/day).

As was mentioned in the introduction, the MoE concept is very universal and useful “toxicological tool” that can be applied to impurities that are both genotoxic and carcinogenic, irrespective of their origin [16]. It has been assumed that the value of MoE of 10,000 (or higher) is considered of low concern from a HHRA point of view with respect to the carcinogenic effect [16]. The justifications for using this approach were (1) documentation in scientific literature for application of this concept for different herbal products (e.g., [17]) and (2) the possibility of applying to children and adolescents as a specific group of the toxicologically relevant population in our case. 

**Figure 1:**
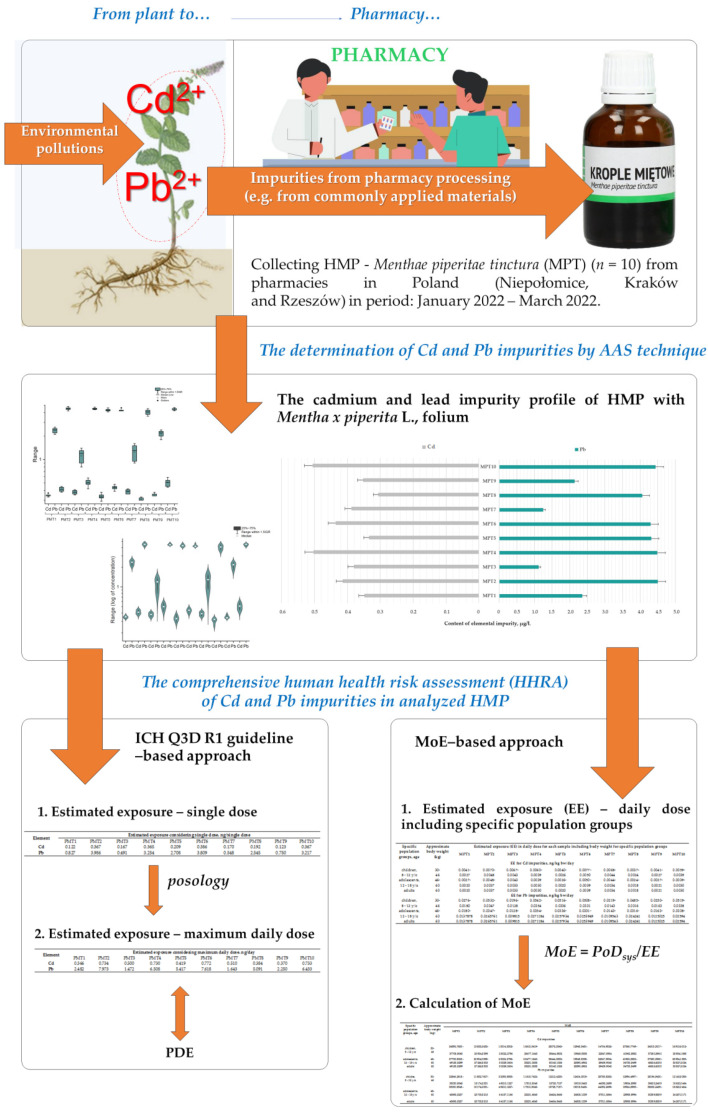
The graphic summary of the applied strategies in comprehensive human health risk assessment (HHRA) of Cd and Pb impurities in HMP with *Mentha × piperita* L., folium available in Polish pharmacies.

The graphic summary of the applied strategies in our comprehensive HHRA of Cd and Pb impurities in analyzed HMP with *Mentha × piperita* L., folium is shown in Figure 1.

## 3. Results

### 3.1. The Cadmium and Lead Impurity Profile of HMP with Mentha × piperita L., folium Available in Polish Pharmacies

**Figure 2:**
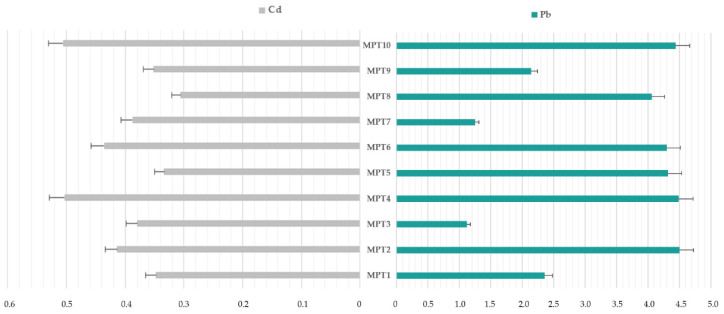
The elemental impurities profile for Cd and Pb in investigated herbal medicinal products with *Mentha × piperita* L., folium (PTM1–PTM10).

As was mentioned in the introduction, the aim of our study is not the determination of chosen heavy metals (Cd and Pb) but the comprehensive HHRA of these impurities in HMP with *Menthae piperitae tinctura* (*Mentha × piperita* L., folium) available in Polish pharmacies. However, the first and obligatory steps for this purpose are acquisition, analysis, and processing of base data (raw results). In these kind of studies (elemental impurities), presentation data as impurity profiles for each element, as shown on Figure 2, are very useful.

Additionally, the descriptive statistics of Cd and Pb impurities levels in analyzed HMP with *Mentha × piperita* L., folium are presented in Table 3.

**Figure 3:**
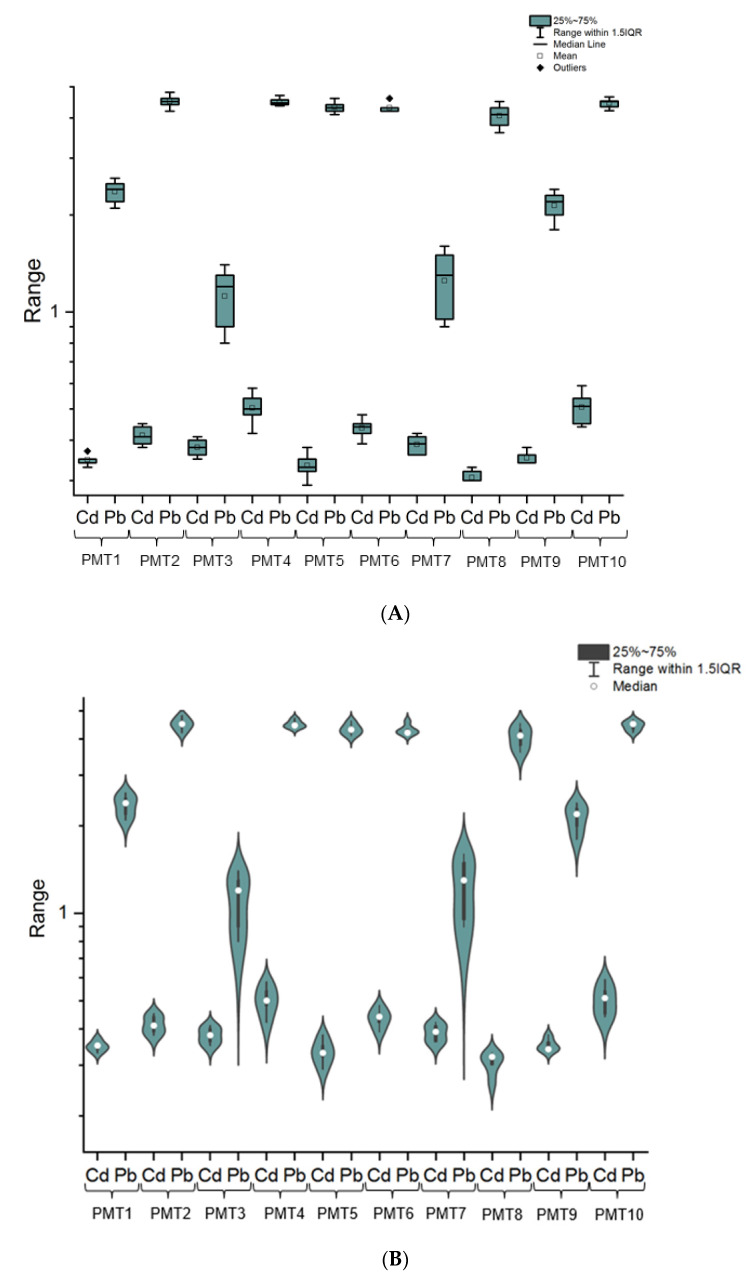
(**A**) The range of Cd and Pb impurities levels in all investigated herbal medicinal products with *Mentha × piperita* L., folium (PTM1–PTM10) as the plotbox (log scale). (**B**) The violin plot showing values of Cd and Pb impurities level (log scale) in all investigated herbal medicinal products with *Mentha × piperita* L., folium (PTM1–PTM10).

Additionally, the plotbox showing ranges of levels (log scale) and violin plot showing levels (log scale) of all investigated impurities are shown in Figure 3A and Figure 3B, respectively.

Taking into account the fact that the main source of contamination in the analyzed samples are probably environmental pollutants and the fact that there is a probability that the raw materials for the production of phytopharmaceuticals may come from a similar source, the ratio of Pb to Cd (Pb/Cd) was calculated for each of the samples (PMT1–PMT10). The obtained results are presented as a second part of Table 3. The analysis of this “indicator” for each HMP should allow for a presumption of similar pollution sources.

### 3.2. The HHRA of Cd and Pb Impurities Considering ICH Q3D R1 Guideline-Based Approach

Based on recommendation from ICH Q3D R1 guideline about elemental impurities in final drugs (considerations only for adults), the appropriate estimation of exposure, including single dose and in daily dose, is necessary. Hence, based on worst-case scenario (WCS), i.e., maximum doses based on known posology for adults (see Table 1) and obtained raw results for Cd and Pb impurities (Figure 2 and Figure 3A,B and Table 3), the exposure in a single-dose (ng/single dose) and maximum daily exposure of investigated elemental impurities (ng/day) were estimated. In this estimation, we considered the experimental fact that approximately 62 drops are equal to 1 g of tincture. The obtained results are summarized together for better readability in Table 4.

### 3.3. The HHRA of Cd and Pb Impurities Considering MoE-Based Approach

As was mentioned in the introduction, considering the fact that investigated phytopharmaceuticals are also applied in children and adolescents (based on posology from each manufacturer—see Table 1), an MoE-based strategy is desirable for a comprehensive HHRA. For this purpose, the values of daily exposure to a product (ng/kg bw/day) were estimated based upon the posology and the average weight of the specific population groups: children (8–12 y/o), adolescents (12–18 y/o), and adults. For this purpose, the estimation of Cd and Pb in daily dose, depending on age and body weight for each population group (based on WHO growth standards [23]), was carry out. The obtained results of a daily dose of Cd and Pb depending on specific population groups in analyzed samples (ng/kg bw/day) are summarized in Table 5.

The last step in this approach is calculation of MoE according to Equation (1). For this purpose, values of EE are calculated in the last section (Table 6). The remaining issue is the choice of the appropriate point of departure (POD_sys_). In most cases, the values of historical NOAEL or BMDL_10_ values from oral animal studies are applied for these purposes. However, for cadmium, several oral exposure studies of cadmium in animal studies (rats and mice) showed no evidence of carcinogenicity [1]. Hence, the key point for HHRA by oral route is renal toxicity of Cd [24], and minimal risk level (MRL) as 0.1 µg/kg bw/day [19] should be applied as POD_sys_. On the other hand, considering for Pb the integrated exposure uptake biokinetic (IEUBK) model [20,25] based on the assumption of 100% absorption, the oral intake of lead is 5.0 μg/day. Additionally, dietary Pb exposure was found to be about one-third lower than previously estimated with mean levels for the four older age groups not exceeding the BMDL_01_ and BMDL_10_ of 1.50 and 0.63 μg/kg bw/day established for cardiovascular and nephrotoxicity effects in adults [21]. Hence, BMDL_10_ (0.63 μg/kg bw/day) should be applied as POD_sys_. The calculated values of MoE depending on specific population groups in analyzed HMP are summarized in Table 6.

## 4. Discussion

### 4.1. The Cadmium and Lead Impurity Profile of HMP with Mentha × piperita L., folium Available in Polish Pharmacies

As already mentioned many times, the aim of this work was not to determine Cd and Pb levels; however, when analyzing the obtained baseline results needed for HHRA, some conclusions can be drawn despite the minimal level of data. Moreover, due to pioneering data (this article is the first study about heavy metals impurities level in final HMPs with *Mentha × piperita* L., folium available in European pharmacies), they can be used by other scientists on aspects of regulatory toxicology (statistics, guides, EMA monographs for HMP, etc.).

Analysis of the raw results indicates that Cd and Pb impurities were present in all analyzed HMP with *Mentha × piperita* L., folium (PTM1–PTM10) available in Polish pharmacies. In general, impurities were present approximately below 4.5 µg/L (in the range of 0.305 to 4.492 µg/L). There is no doubt that in all samples, Pb impurities (mean = 3.407 µg/L) were greatly higher than Cd impurities (mean = 0.397 µg/L); Pb levels were approximately eight time higher (8.37) than cadmium levels (the ratio Pb/Cd were in the range of 2.95 to 13.27). 

The cadmium impurities content was similar and at a relatively low level in all investigated HMP—Figure 3A,B (difference between the highest and lowest level: 0.201 µg/L; Table 3). The highest level of Cd was in PTM4 (0.506 µg/L ± 0.08), and the lowest level was in sample PTM8 (0.306 µg/L ± 0.06). Based on the literature review, it should be stated that no studies on the content of cadmium impurities in this type of pharmaceuticals products or similar matrices have been published so far. There are only indications as to the raw materials showing the presence of Cd [21,22], which was the justification for conducting this type of research. Therefore, it is not possible to reliably compare the obtained results with other studies. However, all HMP with *Mentha × piperita* L., folium contained levels of Cd impurities below the permissible limit set by FAO/WHO for medicinal herbs and plants in different countries (WHO recommendation, 0.3 mg/kg) [21,26]. Additionally, based on permitted levels for Cd in final pharmaceutical products (oral route of exposure) recommended by ICH Q3D R1 guideline (0.5 μg/g [1]), all investigated HMP met the requirements.

On the other hand, Pb impurities were randomized in all analyzed phytopharmaceuticals (in the range of 1.122 to 4.492 µg/L; Table 3, Figure 3A,B). As in the case of Cd, there is limited literature about Pb levels in investigated samples, which makes it impossible to compare the obtained results, but some reports on lead levels in plant raw materials provide the basis for undertaking this type of research. Notwithstanding, in all cases, levels of this element were below the permissible limit set by FAO/WHO for medicinal herbs and plants in different countries (WHO recommendation, 10 mg/kg) [23]. Additionally, considering the ICH Q3D R1 guideline recommended limits (0.5 μg/g [1]), all of the investigated HMP with *Mentha × piperita* L., folium met the requirements.

Although this is not the direct aim of this publication, the Pb/Cd ratio can be applied to try to link the samples together from an environmental point of view, i.e., if the ratio of Pb/Cd is similar in the concerned samples, it is likely that these products were obtained from the same raw material. It follows that the origin of the raw materials can be “delineated” as a specific toxicological fingerprint (Pb/Cd ratio). In this context, the similarity of the pairs can be noticed: PTM1–PTM-9, PTM2–PTM6, PTM3–PTM7, PTM4–PTM10, and PTM5-PTM8. It should be noted, however, that these are only assumptions and are not substantiated by other information.

### 4.2. The HHRA of Cd and Pb Impurities Considering ICH Q3D R1 Guideline-Based Approach

The estimated daily exposure levels for Cd impurities are similar. As was mentioned earlier, the main point for toxicological assessment of Cd by oral route is renal toxicity [19] and the oral PDE value (5.0 µg/day [1]). Our results (Table 5) indicate that all analyzed HMP are characterized by results extremely lower than PDE value for Cd. 

For Pb impurities, the obtained results of daily exposure (Table 5) were generally variable. Based on the integrated exposure uptake biokinetic (IEUBK) model [20] based on the assumption of 100% absorption, the oral intake of this element is 5.0 μg/day, the PDE value for this element should be 5.0 μg/day [1]. The results of our HHRA approach show that all considered phytopharmaceuticals with *Mentha × piperita* L., folium were characterized by results below PDE value. 

### 4.3. The HHRA of Cd and Pb Impurities Considering an MoE-Based Approach

As was mentioned earlier, because ICH Q3D R1 guideline-based approach only includes adults in the HHRA, a different “toxicological tool” should have been used that would allow other specific populations (children and adolescents) to be included in the comprehensive risk assessment as well. For this purpose, firstly, the values of daily exposure to a product (ng/kg bw/day) were estimated (Table 5) based upon the posology and the average weight of the specific population groups: children (8–12 y/o) and adolescents (12–18 y/o). Secondly, the MoE values were calculated (Table 6). In general, MoE of 10,000 or higher (if it is based on the BMDL_10_ from an animal carcinogenicity study and considering overall uncertainties in the interpretation) would be of low concern from a public health point of view and might be reasonably considered as a low priority for risk management actions. Hence, it has been assumed that the value of MoE of 10,000 (or higher) is considered of low concern from a public health point of view. Despite conservative assumptions, the obtained values of MoE for Cd and Pb in the daily dose for each sample (PTM1–PTM10) were above 10,000, and hence, exposure to these elemental impurities would not cause a health risk based on the MoE-based strategy.

## 5. Conclusions

Obtained raw results fill the gap in the data about Cd and Pb impurities existing in HMP as *Menthae piperitae tinctura* (*Mentha × piperita* L., folium) registered in the EU ( for example, the available pharmaceuticals in Poland). Raw results show that Cd and Pb were present in all analyzed HMP with *Mentha × piperita* L., folium (PTM1–PTM10) available in Polish pharmacies. In all samples, Cd impurities (mean = 0.397 µg/L) were far lower than Pb impurities (mean = 3.407 µg/L); the mean ratio Pb/Cd was approximately 8.37.

The HHRA of Cd and Pb impurities considering the ICH Q3D R1 guideline-based approach made it possible to conclude that all results were below the permissible limit set by FAO/WHO for medicinal herbs and plants in different countries (300 µg/kg for Cd and 10,000 µg/kg for Pb). Additionally, the estimated daily intake of investigated elemental impurities compared to the PDE value confirms all samples’ safety. Hence, it can be summarized that all investigated HMP as *Menthae piperitae tinctura* (*Mentha × piperita* L., folium) registered in the EU (available in Poland) meet the standards of the ICH Q3D R1 guideline for elemental impurities in final pharmaceuticals.

On the other hand, the second approach based on the MoE strategy indicated that the obtained values of MoE for Cd and Pb in the daily dose for each samples (PTM1–PTM10) were above 10,000, and hence, exposure to these elemental impurities would not cause a health risk for all investigated population groups (children, adolescents, and adults).

The aim of this article as a comprehensive HHRA of Cd and Pb impurities in HMP as *Menthae piperitae tinctura* (*Mentha × piperita* L., folium) was met through a double regulatory strategy. Application of the ICH Q3D R1 guideline-based approach confirmed the safety of HMP w *Mentha × piperita* L., folium available in Polish pharmacies considering requirements for final pharmaceuticals for both elements. Additionally, the MoE-based strategy made it possible to extend our HHRA to specific groups of the population (children and adolescents) crucial from a toxicological point of view. Despite conservative assumptions, the obtained values of MoE for Cd and Pb impurities in the daily dose for each of the phytopharmaceuticals (PTM1–PTM10) were above 10,000; hence, the safety was confirmed for all investigated samples.

## Figures and Tables

**Table 1:** The summary of all analyzed HMP with *Mentha × piperita* L., folium.

Sample	Posology			Density, G/mL	Composition	OTC
	Children (8–12 y/o)	Adolescents (8–12 y/o)	Adults			
MPT1	Orally: 8–10 drops/ 2 times daily	Orally: 10–15 drops/ 3 times daily	Orally: 15–20 drops/ 3 times daily	0.93	Tincture 1:20 *Mentha × piperita* L. folium, extraction solvent ethanol 80–85% *v/v*	Yes
MPT2	Orally: 8–10 drops/ 3 times daily	Orally: 10–20 drops/ 2 times daily	Orally: 20–50 drops/ 2 times daily	0.92	Tincture 1:20 *Mentha × piperita* L. folium, extraction solvent ethanol 90% *v/v*	Yes
MPT3	Orally: 8–10 drops/ 3 times daily	Orally: 10–15 drops/ 3 times daily	Orally: 15–25 drops/ 3 times daily	0.93	Tincture 1:20 *Mentha × piperita* L. folium, extraction solvent ethanol 90% *v/v*	Yes
MPT4	Orally: 8–10 drops/ 2 times daily	Orally: 15–20 drops/ 2 times daily	Orally: 20–40 drops/ 2 times daily	0.90	Tincture 1:20 *Mentha × piperita* L. folium, extraction solvent ethanol 90% *v/v*	Yes
MPT5	Orally: 10 drops/ 2 times daily	Orally: 10–20 drops/ 2 times daily	Orally: 20–35 drops/ 2 times daily	0.91	Tincture 1:20 *Mentha × piperita* L. folium, extraction solvent ethanol 80–85% *v/v*	Yes
MPT6	Orally: 8–10 drops/ 3 times daily	Orally: 10–20 drops/ 2 times daily	Orally: 20–50 drops/ 2 times daily	0.92	Tincture 1:20 *Mentha × piperita* L. folium, extraction solvent ethanol 90% *v/v*	Yes
MPT7	Orally: 8–10 drops/ 3 times daily	Orally: 10–15 drops/ 3 times daily	Orally: 15–25 drops/ 3 times daily	0.93	Tincture 1:20 *Mentha × piperita* L. folium, extraction solvent ethanol 90% *v/v*	Yes
MPT8	Orally: 10 drops/ 2 times daily	Orally: 10–20 drops/ 2 times daily	Orally: 20–35 drops/ 2 times daily	0.91	Tincture 1:20 *Mentha × piperita* L. folium, extraction solvent ethanol 80–85% *v/v*	Yes
MPT9	Orally: 8–10 drops/ 2 times daily	Orally: 10–15 drops/ 3 times daily	Orally: 15–20 drops/ 3 times daily	0.93	Tincture 1:20 *Mentha × piperita* L. folium, extraction solvent ethanol 80–85% *v/v*	Yes
MPT10	Orally: 8–10 drops/ 2 times daily	Orally: 15–20 drops/ 2 times daily	Orally: 20–40 drops/ 2 times daily	0.90	Tincture 1:20 *Mentha × piperita* L. folium, extraction solvent ethanol 90% *v/v*	Yes

Abbreviations: y/o, years old.

**Table 2:** Brief description of instrumental and experimental conditions for determinations of Cd and Pb in HMP with *Mentha × piperita* L., folium.

Parameter(s)		Element							
		Cd				Pb			
Wavelength, nm		228.8				283.3			
Slit width, nm		0.7				0.7			
Lamp current, mA		5				8			
Optimum working range, µg/kg		0.02–0.20				1.0–10.0			
**Time–** **Temperature** **Program**	**Step**	**Temperature,** **°C**	**Ramp,** **s**	**Hold,** **s**	**Gas Flow, mL/Min**	**Temperature,** **°C**	**Ramp,** **s**	**Hold,** **s**	**Gas Flow, mL/Min**
	1	120	10	25	250	120	1	30	250
	2	300	5	15	250	950	10	20	250
	3	1600	0	3	0	1450	0	5	0
	5	2400	1	2	250	2400	1	2	250

**Table 3:** The descriptive statistics of Cd and Pb impurities levels in analyzed HMP with *Mentha × piperita* L., folium.

Elemental Impurity	Minimum, µg/L	Maximum, µg/L	Range, µg/L		Mean, µg/L		Skewness		Kurtosis	
**Cd**	0.305	0.506	0.201		0.397		1.23		2.52	
**Pb**	1.122	4.492	3.370		3.407		0.67		−2.42	
**Ratio Pb/Cd**	MPT1	MPT2	MPT3	MPT4	MPT5	MPT6	MPT7	MPT8	MPT9	MPT10
	6.78	10.87	2.95	8.91	12.93	9.86	3.22	13.27	6.08	8.77

**Table 4:** The estimated values of exposure of Cd and Pb considering single-dose and maximal daily dose for each HMP with *Mentha × piperita* L., folium (PTM1–PTM10) available in Polish pharmacies.

Element	Estimated Exposure Considering Single Dose. Ng/Single Dose									
	PMT1	PMT2	PMT3	PMT4	PMT5	PMT6	PMT7	PMT8	PMT9	PMT10
**Cd**	0.122	0.367	0.167	0.365	0.209	0.386	0.170	0.192	0.123	0.367
**Pb**	0.827	3.986	0.491	3.254	2.708	3.809	0.548	2.545	0.750	3.217
**Element**	**Estimated Exposure Considering Maximum Daily Dose. Ng/Day**									
	**PMT1**	**PMT2**	**PMT3**	**PMT4**	**PMT5**	**PMT6**	**PMT7**	**PMT8**	**PMT9**	**PMT10**
**Cd**	0.366	0.734	0.500	0.730	0.419	0.772	0.510	0.384	0.370	0.733
**Pb**	2.482	7.973	1.472	6.508	5.417	7.618	1.643	5.091	2.250	6.433

**Table 5:** The estimated exposure of Cd and Pb in the daily dose for each sample (PMT1–PMT10) depending on age and body weight for each specific population group (ng/kg bw/day).

**Specific Population Groups, Age**	**Approximate** **Body Weight** **(Kg)**	**Estimated Exposure (EE) in Daily Dose for Each Sample, including Body Weight for Specific Population Groups**									
		**MPT1**	**MPT2**	**MPT3**	**MPT4**	**MPT5**	**MPT6**	**MPT7**	**MPT8**	**MPT9**	**MPT10**
**EE for Cd Impurities, ng/kg bw/day**											
Children, 8–12 y/o	30– 46	0.0041– 0.0027	0.007– 0.0048	0.0067– 0.0043	0.0060– 0.0039	0.0040– 0.0026	0.0077– 0.0050	0.0068– 0.0044	0.0037– 0.0024	0.0041– 0.0027	0.0059– 0.0039
Adolescents, 12–18 y/o	46– 60	0.0027– 0.0020	0.0048– 0.0037	0.0043– 0.0033	0.0039– 0.0030	0.0026– 0.0020	0.0050– 0.0039	0.0044– 0.0034	0.0024– 0.0018	0.0027– 0.0021	0.0039– 0.0030
Adults	60	0.0020	0.0037	0.0033	0.0030	0.0020	0.0039	0.0034	0.0018	0.0021	0.0030
**EE for Pb Impurities, ng/kg bw/day**											
Children, 8–12 y/o	30– 46	0.0276– 0.0180	0.0532– 0.0347	0.0196– 0.0128	0.0542– 0.0354	0.0516– 0.0336	0.0508– 0.0331	0.0219– 0.0143	0.0485– 0.0316	0.0250– 0.0163	0.0519– 0.0338
Adolescents, 12–18 y/o	46– 60	0.0180– 0.0137878	0.0347– 0.0265761	0.0128– 0.009815	0.0354– 0.0271184	0.0336– 0.0257934	0.0331– 0.0253949	0.0143– 0.0109543	0.0316– 0.024241	0.0163– 0.0125025	0.0338– 0.02594
Adults	60	0.0137878	0.0265761	0.009815	0.0271184	0.0257934	0.0253949	0.0109543	0.024241	0.0125025	0.02594

**Table 6:** Margin of exposure (MoE) calculated for Cd and Pb in daily dose for each sample (PMT1–PMT10) depending on age and body weight for each specific population group.

**Specific Population Groups, Age**	**Approximate** **Body Weight** **(Kg)**	**MoE**									
		**MPT1**	**MPT2**	**MPT3**	**MPT4**	**MPT5**	**MPT6**	**MPT7**	**MPT8**	**MPT9**	**MPT10**
**Cd Impurities**											
Children, 8–12 y/o	30– 46	245,927,650– 377,089,063	136,332,652– 209,043,399	150,145,302– 230,222,796	166,155,419– 254,771,643	250,725,543– 384,445,832	129,453,481– 198,495,338	147,049,522– 225,475,934	273,667,749– 419,623,882	243,133,017– 372,803,960	169,136,012– 259,341,885
Adolescents, 12–18 y/o	46– 60	377,089,063– 491,855,299	209,043,399– 272,665,303	230,222,796– 300,290,604	254,771,643– 332,310,838	384,445,832– 501,451,086	198,495,338– 258,906,962	225,475,934– 294,099,045	419,623,882– 547,335,499	372,803,960– 486,266,035	259,341,885– 338,272,024
Adults	60	491,855,299	272,665,303	300,290,604	332,310,838	501,451,086	258,906,962	294,099,045	547,335,499	486,266,035	338,272,024
**Pb Impurities**											
Children, 8–12 y/o	30– 46	228,462,618– 350,309,348	118,527,607– 181,742,331	320,935,583– 492,101,227	116,157,422– 178,108,048	122,124,233– 187,257,157	124,040,519– 190,195,463	287,558,282– 440,922,699	129,944,997– 199,248,995	251,949,430– 386,322,459	121,435,085– 186,200,464
Adolescents, 12–18 y/o	46– 60	350,309,348– 456,925,237	181,742,331– 237,055,215	492,101,227– 641,871,166	178,108,048– 232,314,845	187,257,157– 244,248,466	190,195,463– 248,081,039	440,922,699– 575,116,564	199,248,995– 259,889,994	386,322,459– 503,898,859	186,200,464– 242,870,171
Adults	60	456,925,237	237,055,215	641,871,166	232,314,845	244,248,466	248,081,039	575,116,564	259,889,994	503,898,859	242,870,171

## Data Availability

Not applicable.
